# Prion pathogenesis is unaltered in the absence of SIRPα-mediated "don't-eat-me" signaling

**DOI:** 10.1371/journal.pone.0177876

**Published:** 2017-05-17

**Authors:** Mario Nuvolone, Marta Paolucci, Silvia Sorce, Veronika Kana, Rita Moos, Takashi Matozaki, Adriano Aguzzi

**Affiliations:** 1 Institute of Neuropathology, University Hospital of Zurich, Zurich, Switzerland; 2 Amyloidosis Research and Treatment Center, Foundation Scientific Institute Policlinico San Matteo, Department of Molecular Medicine, University of Pavia, Pavia, Italy; 3 Division of Molecular and Cellular Signaling, Department of Biochemistry and Molecular Biology, Kobe University Graduate School of Medicine, Kobe, Japan; Deutsches Zentrum fur Neurodegenerative Erkrankungen, GERMANY

## Abstract

Prion diseases are neurodegenerative conditions caused by misfolding of the prion protein, leading to conspicuous neuronal loss and intense microgliosis. Recent experimental evidence point towards a protective role of microglia against prion-induced neurodegeneration, possibly through elimination of prion-containing apoptotic bodies. The molecular mechanisms by which microglia recognize and eliminate apoptotic cells in the context of prion diseases are poorly defined. Here we investigated the possible involvement of signal regulatory protein α (SIRPα), a key modulator of host cell phagocytosis; SIRPα is encoded by the *Sirpa* gene that is genetically linked to the prion gene *Prnp*. We found that *Sirpa* transcripts are highly enriched in microglia cells within the brain. However, *Sirpa* mRNA levels were essentially unaltered during the course of experimental prion disease despite upregulation of other microglia-enriched transcripts. To study the involvement of SIRPα in prion pathogenesis *in vivo*, mice expressing a truncated SIRPα protein unable to inhibit phagocytosis were inoculated with rodent-adapted scrapie prions of the 22L strain. Homozygous and heterozygous *Sirpa* mutants and wild-type mice experienced similar incubation times after inoculation with either of two doses of 22L prions. Moreover, the extent of neuronal loss, microgliosis and abnormal prion protein accumulation was not significantly affected by *Sirpa* genotypes. Collectively, these data indicate that SIRPα-mediated phagocytosis is not a major determinant in prion disease pathogenesis. It will be important to search for additional candidates mediating prion phagocytosis, as this mechanism may represent an important target of antiprion therapies.

## Introduction

Prion diseases are invariably fatal, neurodegenerative disorders caused by misfolded and infectious conformers of the cellular prion protein (PrP^C^) termed prions. These diseases are characterized by extracellular deposition of partially protease-resistant PrP aggregates (termed scrapie prion protein, or PrP^Sc^) within the central nervous system (CNS), sometimes in form of amyloid plaques, accompanied by conspicuous neuronal loss and vacuolation and by pronounced astrogliosis and microgliosis [1].

Microglia activation occurs early during prion disease [2–4]. Microglial cells are often found in the vicinity of prion plaques and can phagocytose PrP^Sc^ [5–7]. Of note, microglia ablation or deficiency results in increased PrP^Sc^ deposits and prion titers, increased accumulation of apoptotic cells, aggravated prion-induced neurotoxicity and accelerated disease progression [8,9]. Collectively, these data demonstrate a general protective role of microglia in prion pathogenesis, possibly through phagocytosis of prion-containing apoptotic bodies [1,8,9].

The molecular mechanisms underlying microglia-mediated removal of prion-containing apoptotic cells have not been fully elucidated. One molecule critically implicated in this phenomenon is milk fat globule epidermal growth factor 8 (Mfge8). This protein, secreted by astrocytes within the brain, opsonizes cerebral apoptotic bodies, thereby favoring their removal [10]. In mice, *Mfge8* genetic ablation results in reduced clearance of apoptotic cells, increased PrP^Sc^ levels and prion titers and accelerated prion disease [10]. Of note, the effect of *Mfge8* ablation on survival upon prion inoculation is present only in certain mouse strains, implying the existence of additional, as of yet unknown polymorphic determinants of prion removal [10,11].

We speculated that one such prion removal determinant might be the signal regulatory protein α (SIRPα)–also known as SHPS-1, BIT or CD172a. SIRPα is a transmembrane protein of the immunoglobulin superfamily with a key role in the control of phagocytosis [12,13]. SIRPα is mainly expressed in myeloid cells, including microglia [12–14]. The best characterized binding partner of SIRPα on phagocytic cells is the ubiquitously expressed “don’t-eat-me” signal CD47 at the surface of cognate cells [12,13]. Upon binding with CD47, the cytoplasmic tail of SIRPα is phosphorylated and recruits the src homology-2 (SH2) domain containing tyrosine phosphatase SHP-1, resulting in a negative signal that inhibits phagocytosis [12,13].

SIRPα is highly polymorphic both in mice [15] and in humans [16], with polymorphic residues mainly located within the CD47-binding domain of the protein and impacting on SIRPα-mediated modulation of phagocytosis [16–20]. Binding of CD47 to SIRPα mediates tethering of apoptotic cells to phagocytes [21,22]. Also, the CD47/SIRPα axis plays a critical role in phagocytosis of senescent erythrocytes, which downregulate CD47, and is exploited by different tumors to escape immunosurveillance through upregulation of CD47 [23–27]. In light of this, the CD47/SIRPα axis has become a new, attractive pharmacologic target to fight cancer [25–28].

Although SIRPα is expressed also in the CNS, the neural function of the CD47/SIRPα axis is less well understood. Recent studies show that CD47 is downregulated within multiple sclerosis brain lesions and that myelin phagocytosis in enhanced by blocking CD47 [14]. Moreover, CD47 was suggested to mediate the regulation of amyloid-β plaques phagocytosis in Alzheimer’s disease, even though its role in this context is controversial [29–33]. In light of the key role of SIRPα in mediating the phagocytosis of apoptotic cells and its possible involvement in the clearance of amyloid-β plaques in Alzheimer’s disease, in the present study we set out to investigate the possible involvement of SIRPα in prion pathogenesis *in vivo*.

## Materials and methods

### Ethical statements

Animal care and experimental protocols were performed in accordance with the Swiss Animal Protection Law and with the “Swiss Ethical Principles and Guidelines for Experiments on Animals” under the approval of the Veterinary office of the Canton of Zurich (permits 41/2012, 90/2013). Prion inoculation was performed under isoflurane anesthesia. All efforts were made in order to prevent or minimize animal suffering.

### *In silico* analysis

Expression of *Sirpa* and related genes in different mouse central nervous system cell types under physiologic conditions was assessed taking advantage of a database of RNA-sequencing transcriptome of purified glia, neurons and vascular cells of the cerebral cortex [34,35]. *Sirpa* mRNA expression levels during microglia development and after lipopolysaccharide-induced immune challenge were obtained from an RNA-sequencing-based transcriptome database for mouse microglia cells [35]. The Prion Disease Database was used to evaluate *Sirpa* and related genes expression in the progression of prion pathogenesis [3,36].

### Mice

*Sirpa* mutant mice on a C57BL/6 background (B6.129P2-*Sirpa*^tm1Nog^, [37]), named here as *Sirpa*^mut/mut^, were obtained from RIKEN, Japan and bred in house. Heterozygous mice were crossed to obtain wildtype, heterozygous and mutant littermates used for the present study. C57BL/6J-isogenic *Prnp* knockout mice of the ZH3 line (B6-*Prnp*^ZH3/ZH3^, [38]) were bred in house. Mice were kept in groups of 3–5 in conventional type II cages in a highly hygienic grade facility under a 12 h light/12 h dark cycle (from 7 am to 7 pm) at 21±1°C. Sterilized food (Kliba No. 3431, Provimi Kliba, Kaiseraugst, Switzerland) and water were provided without restriction.

For baseline analysis, three-month-old littermates were anesthetized with ketamine-xylazine and transcardially perfused with PBS-heparin. For prion inoculations, *Sirpa* mutant, heterozygous and wild-type littermates were intracerebrally inoculated with two different doses of 22L mouse-adapted scrapie (30 ul of 10^−3^ an 10^−5^ dilution of the 22L inoculum, containing 8log LD50 of infectious units per ml in 10% homogenate)[39] or with non-infectious brain homogenate (NBH). Clinical assessment and scoring were performed as previously described [40] (S1 Table). Mice were euthanized with CO_2_ and organs were dissected, snap frozen and kept at -80°C before use.

For RNA sequencing at selected time points during prion pathogenesis, C57BL/6J male mice were purchased from Charles River. The mice were kept in a conventional hygienic grade facility, housed in groups of 3–5 in type IIL cages, under a 12 h light/12 h dark cycle (light from 7 am to 7 pm) at 22±1°C, with unrestricted access to sterilized food (Kliba No. 3340, Provimi Kliba, Kaiseraugst, Switzerland) and water. Mice were anesthetized with isofluorane and injected in the right hemisphere with 30 μl of 0.1% of RML6 (passage 6 of Rocky Mountain Laboratory strain mouse-adapted scrapie prions) or of 0.1% of non-infectious brain homogenate (NBH) from CD-1 mice as control. Prion-infected mice were sacrificed at 4, 8, 12, 16, 18 and 20 weeks post-inoculation or when they reached the terminal stage of prion disease. Control mice injected with NBH were sacrificed one week after the last prion-injected mouse reached the terminal stage. Clinical assessment and scoring were performed as previously described [40] (S1 Table). Euthanasia was performed through transcardial perfusion with PBS after deep anaesthesia with ketamine and xylazinium. Brain areas were dissected, snap frozen and kept at -80°C until further processing. Ten percent (w/vol) tissue homogenates were prepared in 0.25 M sucrose in PBS using a Ribolyzer (Bio-Rad).

### RNA-sequencing

RNA-sequencing analysis on cerebelli and hippocampi from prion inoculated and control mice were conducted as previously described [18,38,40].

### Whole genome single-nucleotide polymorphism (SNP) analysis

Whole genome SNP analysis was conducted as previously described [38]. Genomic DNA (gDNA) extracted from tail biopsies of three littermates (*Sirpa* mutant, heterozygous and wild-type mice) was analysed using the Illumina Mouse MD Linkage Panel array which consists of 1449 strain-informative SNP markers spanning the whole genome (at least three SNPs every 5 Mb; Illumina). SNPs measured in each sample were compared with C57BL/6 and 129 reference strains.

### Restriction fragment length polymorphism analysis

For *Sirpa*, the rs108285434 SNP was analysed as previously described [18]. An amplicon polymorphic between B6 and 129 was generated by PCR from mouse gDNA using the following primers: forward 5’-CCGTTCTGAACTGCACTTTG-3’ and reverse 5’-GGGGTGACATTACTGATACGG-3’ with the following cycling conditions: 2 minutes at 94°C and then 35 cycles of 30 seconds at 94°C, 30 seconds at 58°C, 40 seconds at 72°C and then 7 minutes at 72°C. Amplicons were directly digested with AvaI enzyme in the presence of NEB buffer 4 (both from New England Biolabs) for 2 h at 37°C and digestion products were visualized by agarose gel electrophoresis.

For *Mertk*, the rs27446500 SNP was analysed. An amplicon polymorphic between B6 and 129 was generated by PCR from mouse gDNA using the following primers: forward 5’-TTGGGTTTCATCCCATCCCC-3’ and reverse 5’-GGCACACCTAATCCCCAACT-3’ with the following cycling conditions: 2 minutes at 94°C and then 35 cycles of 30 seconds at 94°C, 30 seconds at 58°C, 40 seconds at 72°C and then 7 minutes at 72°C. Amplicons were directly digested with Hyp188I enzyme in the presence of NEB buffer 4 (both from New England Biolabs) for 2 h at 37°C and digestion products were visualized by agarose gel electrophoresis.

### Western blotting

Ten percent weight/volume homogenates from non-infected cerebelli were prepared in a lysis buffer containing 0.5% 2.5-Dimethoxy-4-chloroamphetamine and 0.5% Nonident P40 in PBS with the addition of cOmplete Mini Protease Inhibitor Cocktail (Roche) and phosSTOP Phosphatase Inhibitor Cocktail (Roche) using stainless steel beads (QIAGEN) and the Tissue Lyser LT (QIAGEN). For prion infected mice, twenty percent weight/volume cerebelli were homogenized in 0.25M sucrose in PBS using a Ribolyzer (Bio-Rad). To detect partially PK-resistant PrP, proteins were digested with PK (Roche) at a final concentration of 25 μg/ml for 30 minutes at 37°C. Total protein concentration was then measured in each sample performing the BCA protein assay (Thermo Fisher Scientific) according to manufacturer’s instructions. Protein homogenates added with NuPAGE LDS sample buffer (Invitrogen) and β-Mercaptoethanol as reducing agent were separated in 12% Bis-Tris gels (Thermo Fisher Scientific) in MES running buffer and transferred onto PVDF or Nitrocellulose membranes (iBlot Transfer Stacks, Thermo Fisher Scientific) using the iBlot Dry Blotting System (Thermo Fisher Scientific), according to manufacturer’s instructions. Precision Plus Protein Dual Color Standards (Bio-Rad laboratories) and MagicMark XP standards to Antibodies (Invitrogen) were loaded into the gel as molecular markers. To take into account possible gradients in signals from the center to the periphery of the membrane when developing, mice of different genotypes were alternated throughout the gel. For Western blots of PK-digested tissues, mirror blots from the same homogenates (omitting the PK-digestion step) were run in parallel, decorated with POM1 and re-probed for actin.

The following antibodies were used: mouse anti-PrP monoclonal antibody POM1 generated in-house (400 ng/ml) [41], anti-actin monoclonal antibody (1: 7000, clone C4, Chemicon), SIRPα C-terminal region antibody (1:1 000; Aviva System Biology) and anti IBA1 antibody (500 ng/mL, Wako) as primary antibodies. Goat anti-mouse HRP-conjugated (1:10 000; Invitrogen) and Goat anti-rabbit IgG HRP-conjugated (1:10 000; Jacksonimmuno) as secondary antibodies. Membranes were blocked in PBS containing 0.1% (vol/vol) Tween 20 (PBST) and TopBlock 5% while the antibody solutions were done in PBST TopBlock 1%. For SIRPα C-terminal region investigation, fat-free milk was used to replace TopBlock. Blots were developed using Luminata Western HRP Substrates (Merck Millipore) and visualized either using the Stella detector (Raytest) or using the Fujifilm LAS-3000 detector. Image levels were adjusted uniformly using Photoshop.

### PrP^C^ Elisa

PrP^C^ levels were quantified by POM1/POM2 sandwich ELISA with minor modification with respect to the previously described protocol [41]. Briefly, a 96-well plate was coated with 400 ng/ml of POM1 capturing antibody and subsequently blocked with PBST 5% TopBlock. Samples were diluted in PBST 1% TopBlock and incubated for two hours before the addition of biotinylated POM2 detection antibody (200 ng/ml). The plate was incubated with HRP-conjugated Avidin (1: 1000; Pharmingen) before the addition of chromogen 3,3′,5,5′-Tetramethylbenzidin (Thermo Fisher Scientific). Sulfuric acid was then used to stop the reaction after 5 minutes from the addition of chromogen.

### Histology and Immunohistochemistry

Formalin-fixed infected brain tissues were treated with concentrated formic acid in order to inactivate prions and were then embedded in paraffin. After deparaffinization with graded alcohols and antigen unmasking procedures in EDTA-based buffer CC1, immunostainings were carried out on brain tissue slices on a NEXES immunohistochemistry robot (Ventana Instruments). The following antibodies were used: IBA1 (1:1000, Wako) and SAF84 (1:200, SPI bio). SAF84 staining was preceded by incubation with protease 2 (Ventana) for 16 min. Haematoxylin and eosin (H&E) staining was performed according to standard procedures. Brain tissues from mice with different genotypes and inoculated with different doses of prions were processed in parallel, together with brain tissue from NBH-injected controls. Slides were scanned using the NanoZoomer scanner (Hamamatse Photonics) and visualized using the NanoZoomer Digital Pathology System (NDPview; Hamamatsu Photonics). Vacuoles and microglia quantifications were performed as previously described [39] in mouse cerebellum granular layers on H&E- and IBA1-stained sections. The regions of interest were drawn on a Digital Image Hub (Leica Biosystems) and the operator was blind to both mouse genotypes and type of inoculum during the entire investigation. The number and the percentage of the structures of interest over the total area were detected using in-house developed software and manual correction of the identified structures was then carried out by the operator. C++ language and OpenCV library were utilised to develop the computational algorithm [39].

### Statistical analysis

One-way ANOVA was used in order to analyze statistically significant differences between the means of groups, and Bartlett’s test was used to evaluate samples equal variances, as appropriate. ANOVA was followed by Bonferroni’s or Tukey’s multiple comparison test or by unpaired t test. Survival times and comparison between the three different genotypes were evaluated using the Kaplan-Mayer method. GraphPad Prism was used for the analysis and the significance level α was set to 0.05 (5%).

## Results

### Expression of *Sirpa* and related genes in the CNS under physiologic conditions

**Fig 1:**
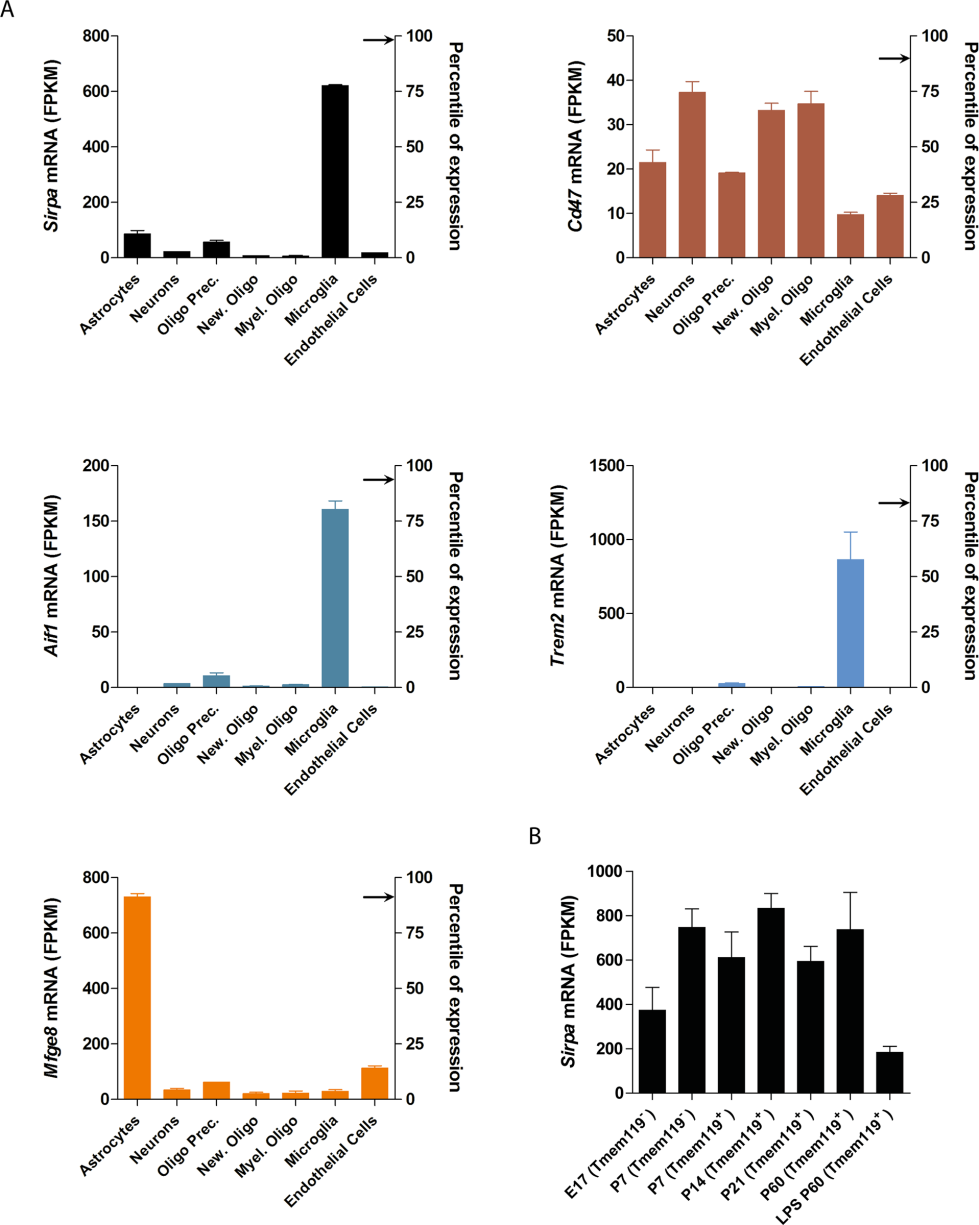
*Sirpa*, *Cd47*, *Aif1*, *Trem2* and *Mfge8* mRNA expression in different CNS cell types. **A** Expression levels of mouse *Sirpa*, *Cd47*, *Aif1*, *Trem2* and *Mfge8* transcripts in different CNS cell populations as assessed by RNA-sequencing of highly purified cells. The left ordinate indicates absolute level of expression whereas the right ordinate shows the percentile expression of the gene with respect to all mouse genes in the CNS. The arrows indicate the percentile values for each gene (*Sirpa*: 98^th^, *Cd47*: 94^th^, *Aif1*: 94^th^, *Trem2*: 83^th^, *Mfge8*:91^th^). Data are obtained from the Brain RNA-seq database [34]. Olig. Prec: Oligodendrocytes Precursors; New. Oligo: Newly formed Oligodendrocytes; Myel. Oligo: Myelinating Oligodendrocytes. Olig. Prec. show 5% of microglial contamination **B** *Sirpa* expression levels during microglia development from embryonic day 17 (E17) to post-natal day 60 (P60) and after immune challenge with lipopolysaccharide (LPS). Tmem119 is a stable microglial marker developmentally regulated. These data are obtained from the Brain RNA-seq database [35]. **A-B** Bars indicate mean values whereas error bars represent standard deviation (n = 2 =). FPKM: fragments per kilobase of transcripts per million mapped reads.

We first analyzed *Sirpa*, *Cd47*, *Aif1* (encoding IBA1), *Trem2* and *Mfge8* mRNA expression levels in glial cells, neurons and vascular cells of the cerebral cortex in mice by interrogating the RNA-sequencing-based transcriptome database [34]. *Sirpa* was significantly enriched in microglia/macrophages (P < 0.001, Tukey’s Multiple Comparison with all other CNS cell population), similarly to *Aif1* and *Trem2* (Fig 1A). There was no individual cell population with a significant upregulation of *Cd47* expression with respect to all the other cell types, whereas *Mfge8* was mainly expressed in astrocytes (P < 0.001, Tukey’s Multiple Comparison with all other CNS cell population; Fig 1A), in line with previous observations [10]. Of interest, *Sirpa* and *Cd47* were among the most highly expressed genes within the CNS, locating at the 98^th^ and the 90^th^ percentile of expression amongst all murine genes at this site (Fig 1A).

We further investigated how *Sirpa* expression levels varied during microglia development from embryonic day 17 (E17) to post-natal day 60 (P60) and after an immune challenge [35]. We observed that *Sirpa* is already expressed at E17 and its expression further increases postnatally. However, lipopolysaccharide-induced immune challenge at P60 leads to a significant drop in *Sirpa* expression levels on cultured primary microglia (P = 0.0319, Unpaired t test) (Fig 1B).

### Expression of *Sirpa* and related genes in the CNS during prion pathogenesis

**Fig 2:**
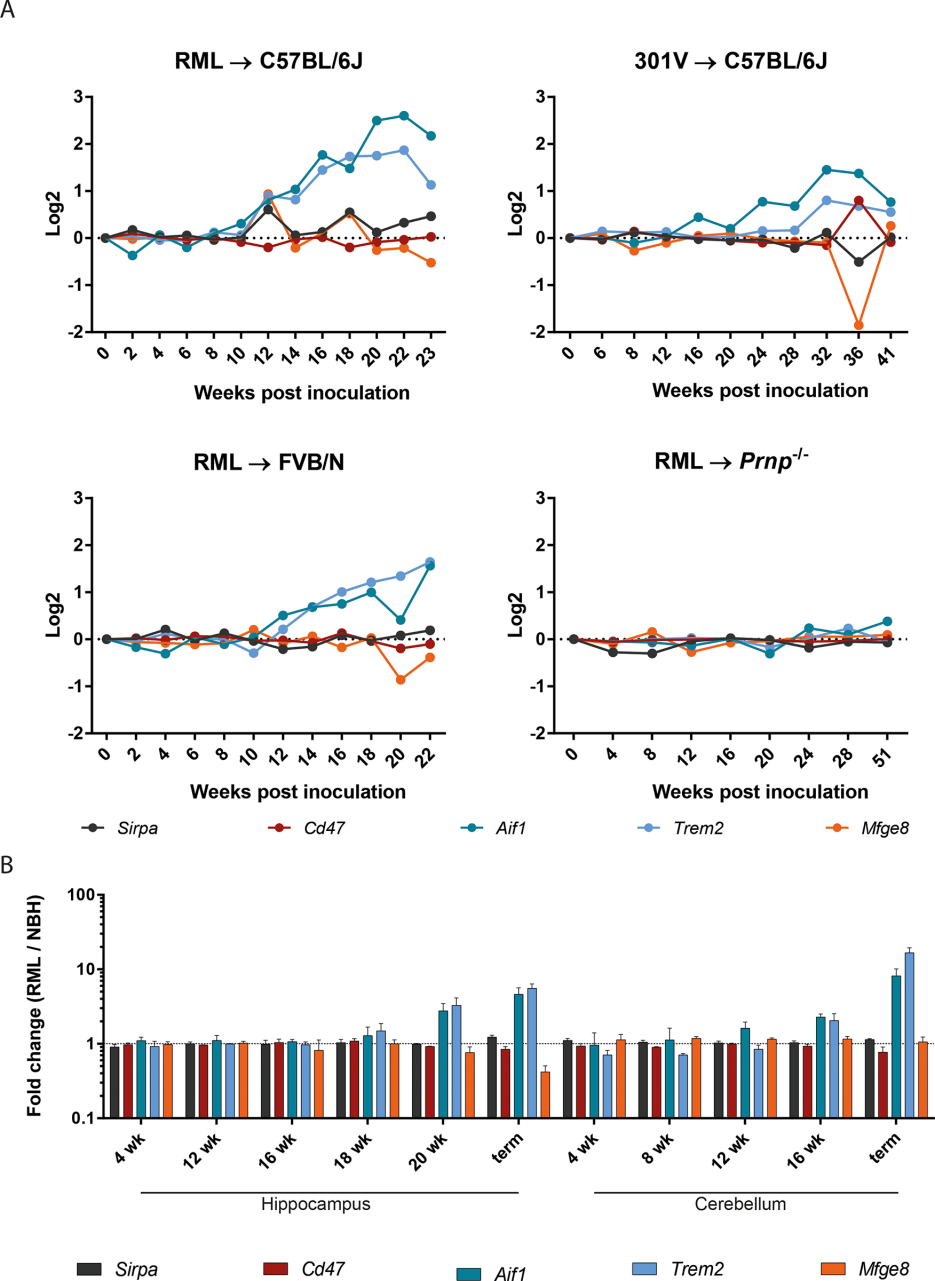
*Sirpa*, *Cd47*, *Aif1*, *Trem2* and *Mfge8* mRNA levels during experimental prion pathogenesis. **A** Gene expression profile at different time points during the development of prion disease. Microarray analysis was conducted on a combination of different mouse and prion strains: RML → C57BL/6J; 301V → C57BL/6J and RML → FVB/Ncr. RML→ Prnp^*-/-*^ was used as control, these mice not being susceptible to the disease. Data were obtained from the Prion Disease Database [3,36]. **B** *Sirpa*, *Cd47*, *Aif1*, *Trem2* and *Mfge8* mRNA levels in hippocampus and cerebellum of RML-inoculated mice (n = 3) compared to NBH-injected controls (n = 3). Bars represent mean and standard deviation of transcript fold change expression between brains of RML-inoculated mice and NBH-injected controls at different time points (expressed as weeks, wk) during the progression of prion disease, or at terminal stage (term). Dashed line indicates equal levels between RML-inoculated mice and NBH-injected controls (fold change 1).

We next investigated *Sirpa* mRNA profile in brain of mice during the progression of prion disease by taking advantage of the Prion Disease Database [36]. The temporal pattern of *Sirpa* levels was compared with the pattern of *Cd47*, *Aif1*, *Trem2* and *Mfge8* transcripts. This analysis was conducted including different combinations of mouse and prion strains and results were compared with baseline data obtained from prion-inoculated *Prnp* knockout mice not susceptible to prion infection [36]. *Sirpa* and *Cd47* mRNA levels showed some fluctuations during the progression of prion diseases, with some degree of variation based on mouse and prion strain combination, and the same occurred also to *Mfge8* levels (Fig 2A). Conversely, progressive upregulation of *Aif1* and *Trem2* transcripts was systematically observed in prion pathogenesis (Fig 2A).

In addition, we analysed *Sirpa*, *Cd47*, *Aif1*, *Trem2 and Mfge8* mRNA levels selectively in hippocampus and cerebellum at different time points during the progression of prion disease using RNA sequencing. Again, we noticed that *Aif1* and *Trem2* transcripts were upregulated in the late and terminal stages of the disease, both in the hippocampus and in the cerebellum, whereas *Sirpa* and *Cd47* mRNA levels were stable during prion pathogenesis (Fig 2B, S2 Table).

### Characterization of *Sirpa* mutant mice

**Fig 3:**
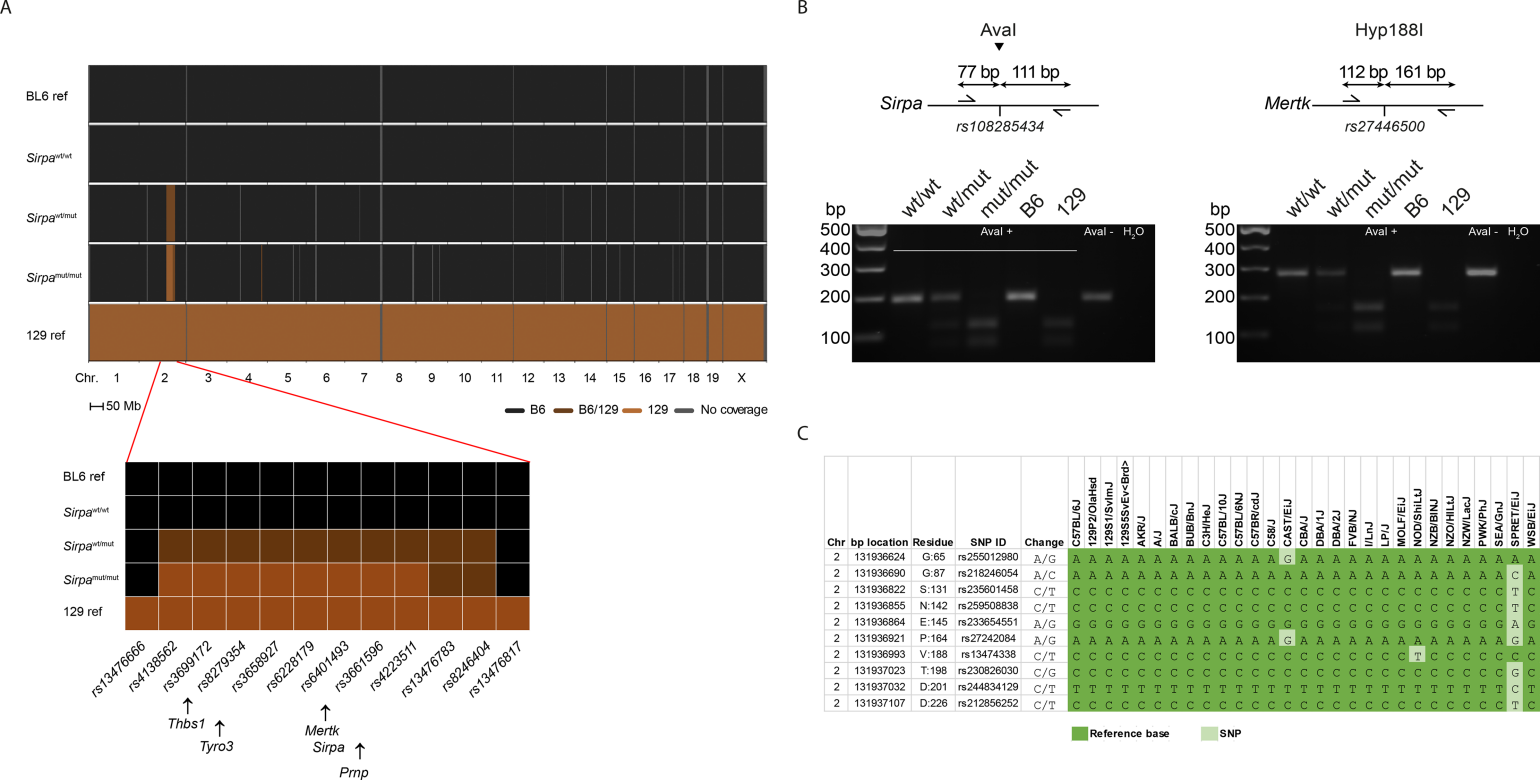
Genetics of *Sirpa* mutant mice. **A** Whole genome SNPs analysis. Data are from three individual littermates (*Sirpa*^wt/wt^, *Sirpa*^wt/mut^, *Sirpa*^mut/mut^). Among 1449 single SNPs tested, only 863 mapped SNPs concordant for C57BL/6 substrains and informative between C57BL/6 and 129 strains are displayed. SNPs are mapped based on their physical location. BL6 ref. and 129 ref.: reference data for C57BL/6 and 129 strains, respectively. Bottom panel: magnification of chromosome 2 region harboring 129-derived genetic material in *Sirpa*^mut/mut^ mouse. This region is comprised between SNP marker rs13476666 (position 101217024) and rs13476817 (position 144125632). Arrows indicate the physical position of *Thbs1* (118111876–118127133), *Tyro3* (119797733–119818104), *Mertk* (128698956–128802894), *Sirpa* (129592835–129632228) and *Prnp* (131909928–131938429). **B** RFLP analysis within the *Sirpa* (left) and *Mertk* (right) loci discriminating *Sirpa*^wt/wt^ (wt/wt; undigested amplicon), *Sirpa*^wt/mut^ (wt/mut; both digested and undigested amplicons) and *Sirpa*^mut/mut^ (mut/mut; digested amplicon). Primers location, restriction site, restriction enzyme and expected amplicon sizes are represented on the top of each gel image. Control: undigested sample. **C** SNPs within *Prnp* in a panel of inbred strains of the laboratory mouse. Each SNP site, in the rows, is denoted with its physical location on chromosome 2 (Chr 2), its code and, for SNPs within the *Prnp* coding sequence, the corresponding residue. Colors indicate whether the sequence of a specific strain, in the columns, coincides with the reference C57BL/6J sequence (dark green) or not (light green). Sequence data were retrieved from the Mouse Phenome Database.

To evaluate the possible involvement of SIRPα in prion disease, we analysed *Sirpa* mutant mice expressing a truncated form of the protein which is unable to mediate intracellular signaling [37]. *Sirpa* gene targeting was performed in 129-derived embryonic stem cells and targeted mice were repeatedly backcrossed to C57BL/6 [37], thereby generating congenic B6.129 mice. Accordingly, these mice are expected to harbor a 129-derived portion of chromosome 2, comprising the targeted locus and neighboring regions in linkage disequilibrium with it, in the context of an otherwise C57BL/6 genome [18,42]. Indeed, whole genome SNP analysis revealed that 98.58%, 99.30% and 100% of all analyzed SNPs were compatible with C57BL/6 genome in *Sirpa*^mut/mut^, *Sirpa*^wt/mut^ and *Sirpa*^wt/wt^ mice, respectively (Fig 3A). Non-C57BL/6 SNPs, compatible with the 129 genome, were observed in *Sirpa*^mut/mut^ and *Sirpa*^wt/mut^ and almost exclusively on chromosome 2, in a region comprised between the SNP marker rs13476666 (Chr2:101217024) and rs13476817 (Chr2:144125632) (Fig 3A). Of interest, besides the targeted *Sirpa* locus, this region also contained *Prnp* and other genes known to impact on phagocytosis, such as *Mertk*, *Thbs1* and *Tyro3* (Fig 3A), in line with previous reports of close genetic linkage among these genes [18].

To further verify the 129-origin of this genomic region, we performed restriction fragment length polymorphism (RFLP) analysis within the *Mertk* locus, focusing on a SNP informative between C57BL/6 and 129 strains. As a control, we also investigated an informative SNP in the *Sirpa* locus, which is expected to be of 129 type in the *Sirpa*^mut/mut^ mice due to the origin of the embryonic stem cells used for gene targeting [37]. These analyses confirmed that *Sirpa*^mut/mut^ mice display 129-derived SNPs in both *Mertk* and *Sirpa* loci (Fig 3B).

Since polymorphisms in *Prnp* sequence can significantly influence prion disease incubation time [43,44], we first verified which *Prnp* allele is present in 129 strains of the laboratory mouse. For this purpose, we retrieved sequence information of the *Prnp* locus from the Mouse Phenome Database for different 129 substrains, and compared it with C57BL/6 sequence as reference and with published sequences of different *Prnp* allelotypes. This analysis showed that all analyzed 129 substrains of the laboratory mouse carry the *Prnp*^a^ allele, the same as C57BL/6J reference (Fig 3C). This excluded the possibility that different *Prnp* alleles might represent a systematic confounder when comparing *Sirpa*^mut/mut^, *Sirpa*^wt/mut^ and *Sirpa*^wt/wt^ mice.

**Fig 4:**
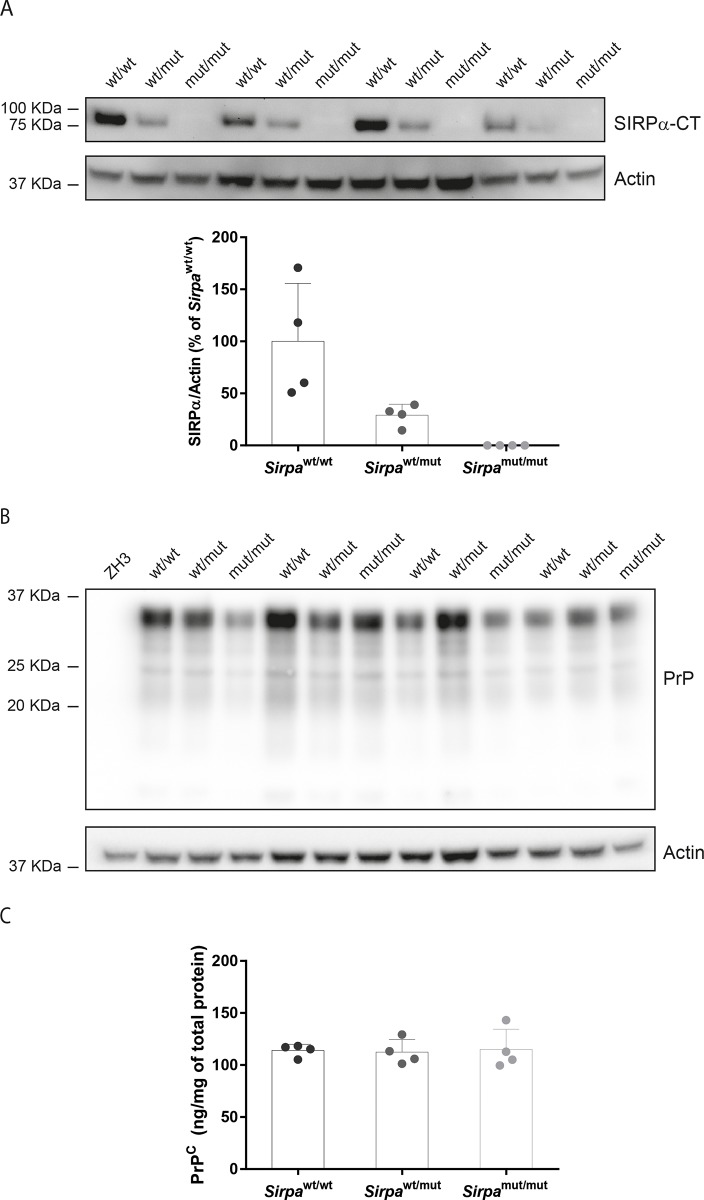
SIRPα and PrP^C^ levels in *Sirpa* mutant mice. **A** Western blot analysis of cerebellar homogenates from *Sirpa*^wt/wt^ (wt/wt), *Sirpa*^wt/mut^ (wt/mut) and *Sirpa*^mut/mut^ (mut/mut) mice using SIRPα-CT antibody and actin as loading control. Each lane denotes a mouse. The bottom panel shows the quantification of SIRPα levels normalised to actin. Each dot denotes a mouse. Bars indicate mean value whereas error bars represent standard deviation. **B** PrP^C^ expression levels were assessed by Western blotting using POM1. Actin was used for loading control. *Prnp*^ZH3/ZH3^ (ZH3) lacking PrP^C^ was used as a negative control. **C** ELISA test to quantify PrP^C^ levels in cerebellar homogenates of *Sirpa*^wt/wt^ (wt/wt), *Sirpa*^wt/mut^ (wt/mut) and *Sirpa*^mut/mut^ (mut/mut) mice. Each dot represents a mouse. Bars indicate mean value whereas error bars represent standard deviation. No significant difference in PrP^C^ levels was observed (P > 0.05, One-way ANOVA).

In B6.129 congenic mice, *cis*-regulatory elements in the 129-derived region flanking the targeted locus with polymorphic variants between C57BL/6 and 129 strains could impact on the regulation of neighboring genes, increasing or reducing their expression levels [38,45]. As PrP^C^ levels are a strong determinant of prion disease incubation [43,46,47], we investigated PrP^C^ expression in the cerebellum of *Sirpa*^mut/mut^, *Sirpa*^wt/mut^ and *Sirpa*^wt/wt^ littermates. As a control, we first verified the expression of SIRPα using an antibody targeting the cytoplasmic tail of the protein to distinguish between the full length and the truncated mutant protein. As expected, we were unable to detect any full length SIRPα in *Sirpa*^mut/mut^ mice and we observed a lower expression of full length SIRPα in *Sirpa*^wt/mut^ with respect to *Sirpa*^wt/wt^ littermates (Fig 4A). We then evaluated PrP^C^ expression. Western blotting using anti-PrP^C^ antibody POM1 showed similar PrP^C^ levels and glycosylation patterns among the three genotypes (Fig 4B). Congruently, sandwich ELISA using anti-PrP^C^ antibodies POM1 and POM2 did not detect any significant difference in PrP^C^ concentration (Fig 4C; P > 0.05, One-way ANOVA). Collectively these results exclude the presence of differences in PrP^C^ brain levels among *Sirpa*^mut/mut^, *Sirpa*^wt/mut^ and *Sirpa*^wt/wt^ mice.

### Effects of *Sirpa* mutation in prion pathogenesis

**Fig 5:**
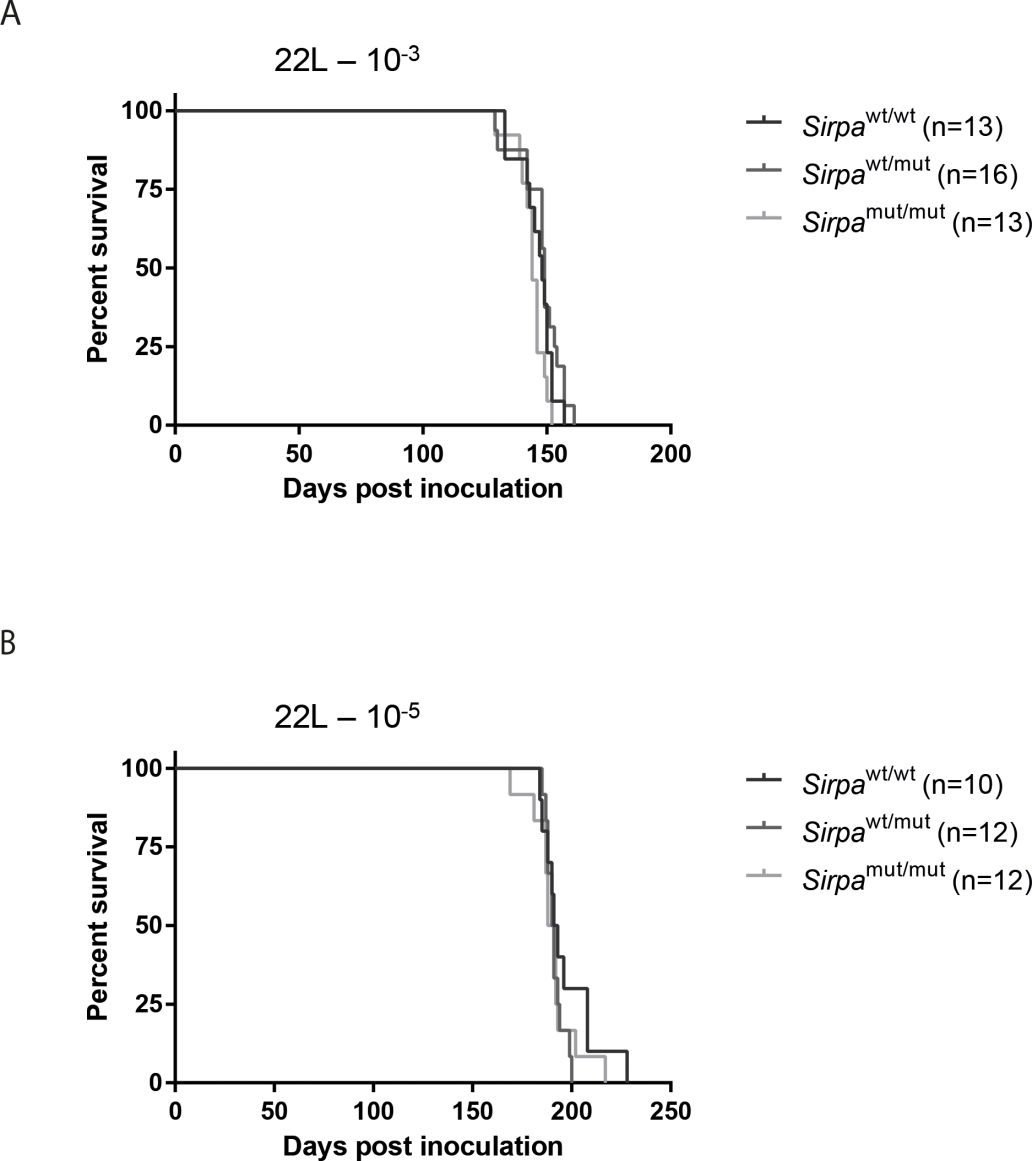
SIRPα mutation does not impact on the progression of prion disease. **A-B** Kaplan-Meier survival curves of, *Sirpa*^wt/wt^, *Sirpa*^wt/mut^ and *Sirpa*^mut/mut^ inoculated intracerebrally with (**A**) 22L 10^−3^ (*Sirpa*^wt/wt^ n = 13, median incubation time 148 days post inoculation (dpi); *Sirpa*^wt/mut^, n = 16, 149 dpi; *Sirpa*^mut/mut^, n = 13, 144 dpi) or (**B**) 22L 10^−5^ (*Sirpa*^wt/wt^, n = 10, 192 dpi; *Sirpa*^wt/mut^, n = 12, 191 dpi; *Sirpa*^mut/mut^, n = 12, dpi 190;). **A-B** No statistically significant difference among genotypes was observed.

Next, *Sirpa*^mut/mut^, *Sirpa*^wt/mut^ and *Sirpa*^wt/wt^ littermates were intracerebrally challenged with rodent-adapted scrapie prions of the 22L strain. Interestingly, there was a trend towards reduced incubation time in mice expressing the mutant SIRPα protein, with median incubation of 144, 148 and 149 days post injection (and mean ± standard error of the mean, SEM, of 144±2, 148±2 and 146±2) for *Sirpa*^mut/mut^, *Sirpa*^wt/mut^ and *Sirpa*^wt/wt^ littermates, respectively (Fig 5A). However, this difference did not reach statistical significance (P = 0.0524, Log-rank test). Conversely, there was no substantial difference in disease incubation after challenge with a lower dose of prions, with median incubation of 190, 192 and 191 days post injection (and mean SEM of 191±3, 191±1 and 197±4) for *Sirpa*^mut/mut^, *Sirpa*^wt/mut^ and *Sirpa*^wt/wt^ littermates, respectively (P = 0.3688, Log-rank test) (Fig 5B).

**Fig 6:**
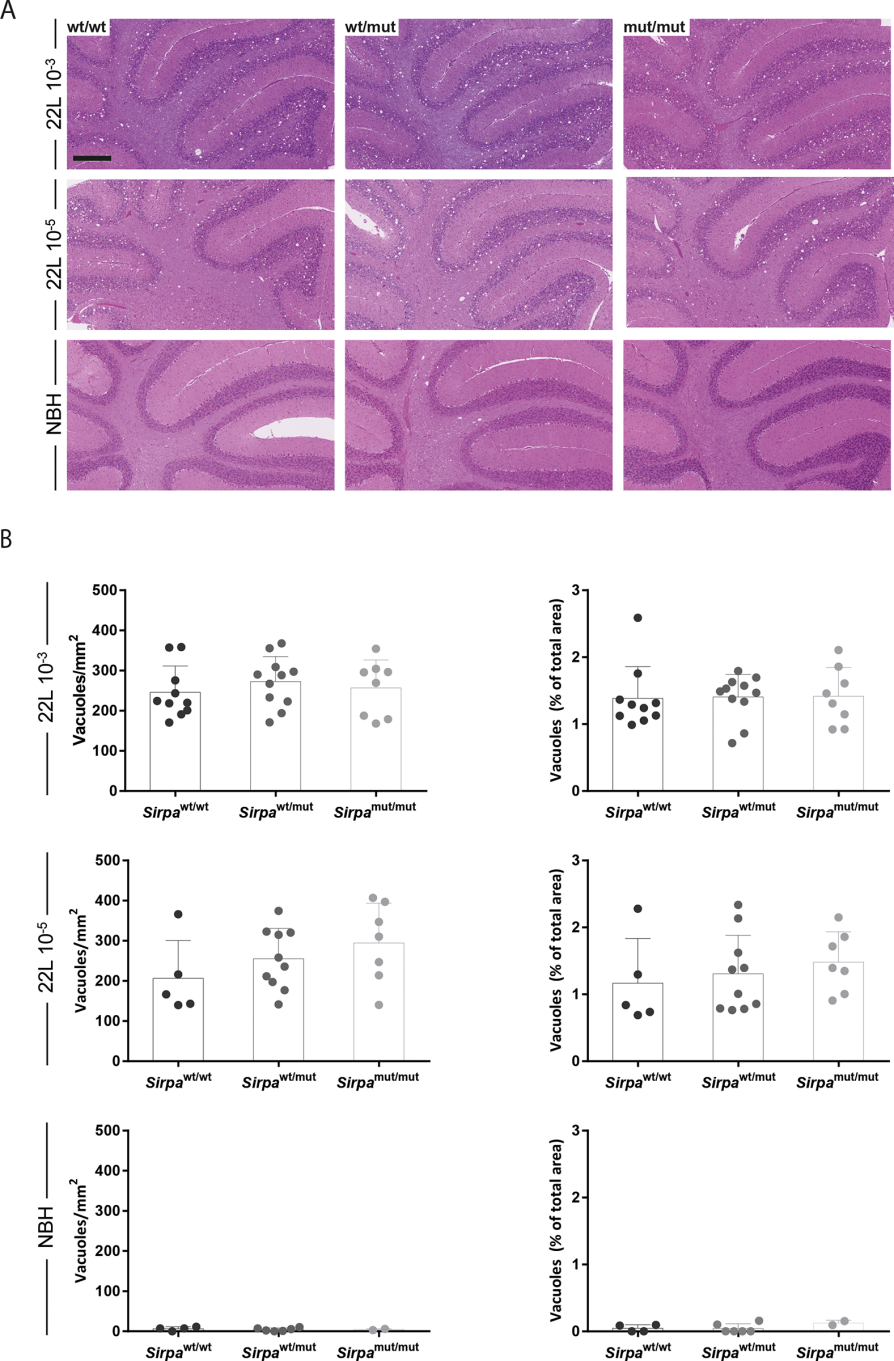
Effects of SIRPα mutation on spongiform changes after prion inoculation. **A** Histological analysis on cerebellar haematoxylin and eosin (H&E) stained slices of *Sirpa*^wt/wt^ (wt/wt), *Sirpa*^wt/mut^ (wt/mut) and *Sirpa*^mut/mut^ (mut/mut) mice inoculated with 22L 10^−3^, 22L 10^−5^ prion doses or injected with NBH. Scale bar 250 μm. **B** The number of vacuoles per square mm of surface (left) and the percentage of area covered by vacuoles (right) were quantified on the same cerebellar H&E-stained sections. Bars indicatemean value whereas error bars represent standard deviation. Each dot represents a mouse. No statistically significant changes were observed among the three genotypes.

Besides disease incubation, we explored whether ablation of a functional SIRPα-dependent signaling would impact on histologic and biochemical hallmarks of prion diseases. We first evaluated the presence of spongiosis. Compared to control mice intracerebrally injected with NBH, prion-inoculated mice showed a prominent vacuolation (Fig 6A), with no significant differences among the three genotypes (P > 0.05, One-way ANOVA) (Fig 6B).

**Fig 7:**
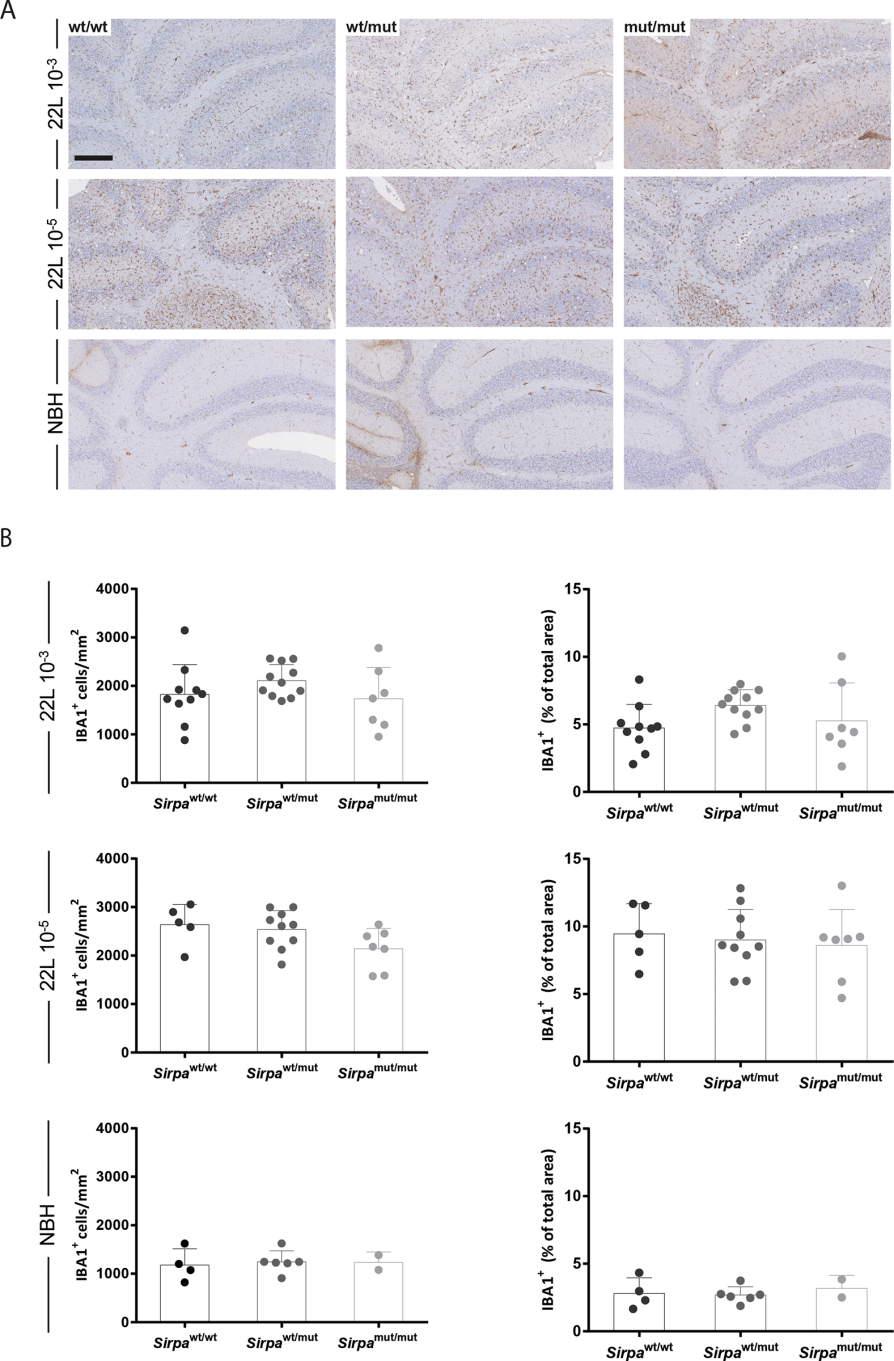
Effects of SIRPα mutation on microglial activation after prion inoculation. **A** Microglial cells were analysed by immunohistochemistry on cerebellar sections stained for IBA1 in *Sirpa*^wt/wt^ (wt/wt), *Sirpa*^wt/mut^ (wt/mut) and *Sirpa*^mut/mut^ (mut/mut) mice inoculated with 22L 10^−3^, 22L 10^−5^ prion doses or injected with NBH. Scale bar: 250 μm. **B** The number of IBA1 positive cells per square mm of surface (left) and the percentage of area covered by IBA1 positive cells (right) were quantified on the same IBA stained cerebellar slices. The percentage of surface occupied by IBA1 stained structures over the total area was measured in selected regions of the cerebellar cortex for each genotype inoculated with 22L prions or injected with NBH. Bars indicatemean value whereas error bars represent standard deviation. Each dot represents a mouse. No statistically significant differences were observed among genotypes.

We next investigated the extent of microgliosis through immunohistochemical staining for IBA1. Again, compared to NBH-injected controls, 22L-inoculated mice showed a remarkable IBA1 immunoreactivity (Fig 7A), with no significant differences among the three genotypes (P > 0.05, One-way ANOVA) (Fig 7B).

**Fig 8:**
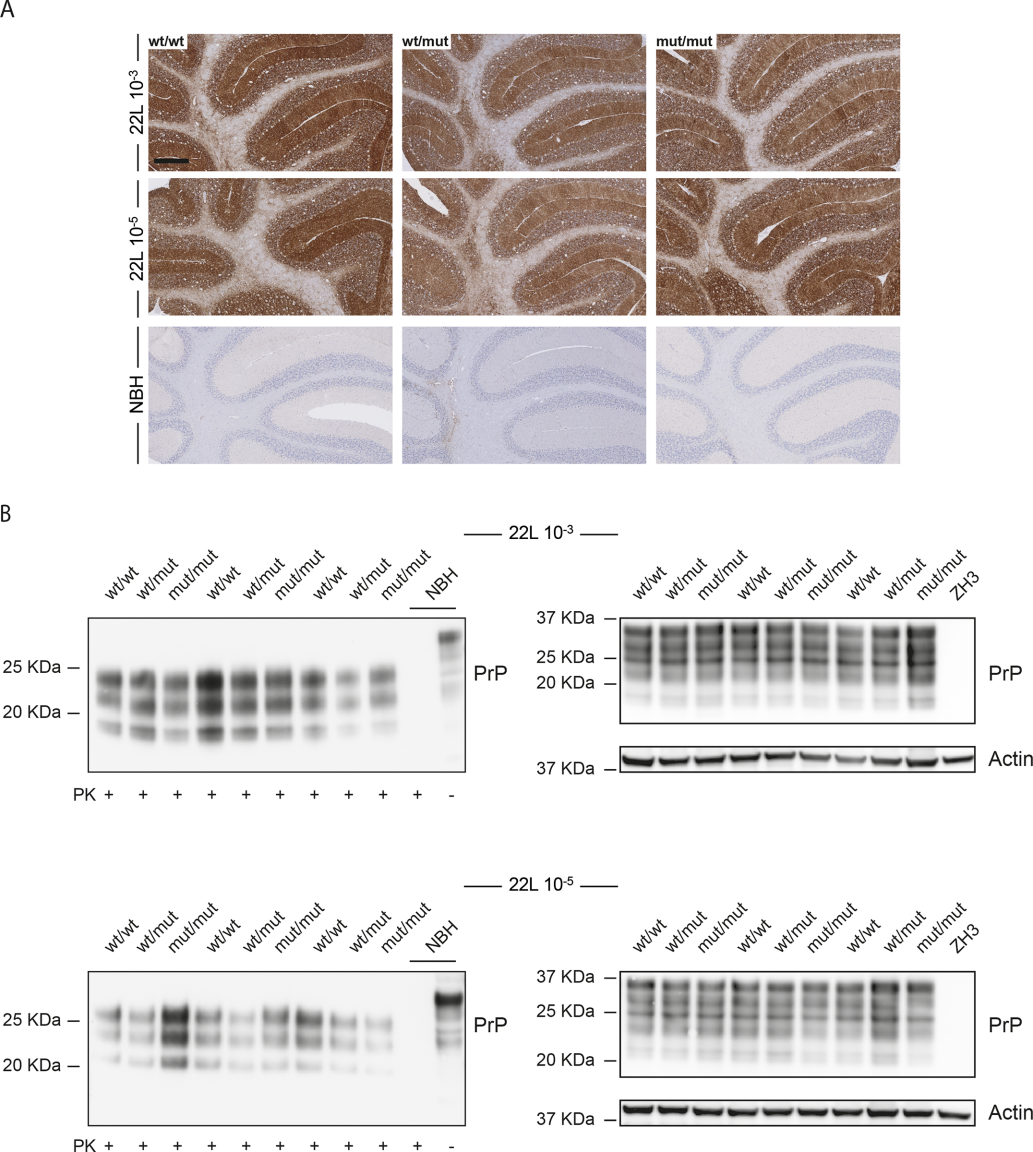
Effects of SIRPα mutation on partially protease-resistant PrP after prion inoculation. **A** Immunohistochemical staining for partially protease-resistant PrP (SAF84) on cerebellar sections of *Sirpa*^wt/wt^ (wt/wt), *Sirpa*^wt/mut^ (wt/mut) and *Sirpa*^mut/mut^ (mut/mut) littermates inoculated with 22L 10^−3^, 22L 10^−5^ prion doses or NBH. Scale bar: 250 μm. **B** Left: Western blot analysis of partially protease K (PK)-resistant PrP in PK digested cerebellar homogenates of *Sirpa*^wt/wt^ (wt/wt), *Sirpa*^wt/mut^ (wt/mut) and *Sirpa*^mut/mut^ (mut/mut) littermates inoculated with 22L prions. Negative control for PK Western blots → cerebellar homogenate of a mouse injected with NBH and digested (PK+) or not digested (PK) with proteinase K. Right: Mirror Western blot with the same samples as in the left blot, but with the omission of the PK digestion step. The blot was decorated first with POM1 (upper image) and then re-probed with an actin antibody (lower image). Negative control for non-PK western blots → *Prnp*^ZH3/ZH3^ (ZH3) lacking PrP^C^ and not susceptible to prion infection. Each lane denotes a mouse.

Finally, we investigated levels of disease-associated PrP accumulation. Immunohistochemical analysis confirmed the presence of partially protease-resistant PrP in the brains of prion-inoculated–but not in NBH-injected–mice (Fig 8A). Similarly, Western blotting of partially PK-resistant PrP showed similar levels of disease-associated PrP among the three genotypes for both series with different prion doses (Fig 8B).

## Discussion and conclusion

Prion diseases are characterized by the deposition of misfolded PrP in the brain associated to neuronal loss, vacuolation and astro- and microgliosis [1]. The mechanisms controlling microglia activation are still unclear. However microglia-mediated phagocytosis of disease-associated PrP deposits is a crucial mechanism of defense deployed by the CNS against prion infection [9].

In this study we focused our attention on the role of SIRPα, a transmembrane protein known to be involved in the negative regulation of phagocytosis [13]. Increasing evidence from previous studies suggested that SIRPα in the brain is involved in the control of microglia-mediated myelin phagocytosis [48] and that CD47, the better characterized ligand of SIRPα, is implicated in microglia-mediated phagocytosis of amyloid-β in Alzheimer’s disease [29–32], even though contradictory data exist [33].

When we analysed *Sirpa* expression levels in the CNS, we found that *Sirpa* was mainly found on microglia. In light of the fact that SIRPα functions as a mediator of phagocytosis, we asked whether SIRPα could also play a role in prion pathogenesis by modulating the phagocytosis of apoptotic cells and PrP^Sc^ deposits engulfment.

During the progression of prion pathogenesis, transcripts expressed by microglia, including *Aif1 –*encoding for IBA1 –and *Trem2*, displayed a significant upregulation, whereas *Sirpa* mRNA levels measured at different time points did not show a specific trend of gene expression profile.

The lack of significant upregulation of *Sirpa* transcripts during the course of prion disease does not exclude a role for SIRPα in prion pathogenesis. For example, also astrocytic secreted-Mfge8, which is known to promote phagocytic engulfment and clearance of PrP^Sc^ [10], did not show any specific tendency of regulation.

To study the role of SIRPα in prion pathogenesis *in vivo*, we analyzed congenic *Sirpa* mutant mice lacking the cytoplasmic part of the protein [37]. The mutated form of SIRPα, not susceptible to phosphorylation, was unable to recruit its effector SHP-1 and mediate the downregulatory don’t-eat-me signal [49]. Studies conducted on *Sirpa* mutant mice in the CNS confirmed that these mice were unable to initiate such negative regulation on phagocytes [48].

*Sirpa* and *Prnp* are both located on chromosome 2 in close proximity (about 2.2 Mb) [15] and they display a strong linkage disequilibrium [18]. A genome-wide SNPs analysis of congenic *Sirpa* mutant mice, generated in 129 embryonic stem cells and backcrossed to C57BL/6 mice, revealed that a significant portion of chromosome 2 consists of 129-derived genomic material, including also *Prnp*. These genetic characteristics of *Sirpa* mutant mice could represent confounding features in the context of our study. First of all, polymorphisms in *Prnp* sequence may influence the incubation time of the disease. However, our analysis of the Mouse Phenome Database shows that 129 strains express *Prnp*^*a*^ allele, the same known to be expressed in C57BL/6 strains, excluding the concern that *Sirpa* mutant and wild-type mice might have a different *Prnp* allele. Secondly, *cis*-regulatory elements with polymorphic variants between C57BL/6 and 129 strains could impact on the regulation of neighboring genes, increasing or reducing their expression levels [38,45]. For this reason, being the timing of prion disease dependent on PrP^C^ expression [43,46], we first evaluated PrP^C^ concentration in the CNS of *Sirpa*^mut/mut^, *Sirpa*^wt/mut^ and *Sirpa*^wt/wt^ littermates. We found no significant difference among the three genotypes. These observations exclude the possibility that different *Prnp* allelotypes or different levels of PrP^C^ expression may act as confounding features in our study. Thirdly, other genes impacting on phagocytosis were identified on chromosome 2. Among these *Mertk*, *Thbs1*, and *Tyro3* display nonsynonymous SNPs between C57BL/6 and 129 strains [18] and they could act as genetic confounders in studies using congenic *Sirpa*^mut/mut^ mice. Indeed, the ablation of SIRPα functions could be balanced or overcome by these polymorphic genes involved in the regulation of phagocytosis. However, numerous reports confirm that macrophages from this line of *Sirpa*^mut/mut^ mice have an increased phagocytic activity [50–52]. In this context, the recent generation of a conditional *Sirpa* knockout mouse line allowing the specific ablation of functional SIRPα will prove instrumental to investigate the consequences of the selective ablation of SIRPα using the cre/loxP system [53].

In *Sirpa*^mut/mut^ mice inoculated with different doses of 22L prions, we were unable to observe significant differences in survival times compared to *Sirpa*^wt/mut^ and *Sirpa*^wt/wt^ littermates. Interestingly, *Sirpa*^mut/mut^ mice displayed comparable degrees of vacuolation and microgliosis to *Sirpa*^wt/mut^ and *Sirpa*^wt/wt^ littermates, showing similar disease progression. Therefore, spongiform changes and microglia reactivity to prion infection were not appreciably modulated by expression of truncated non-functional form of SIRPα instead of the full-length protein.

Similarly, *Sirpa*^mut/mut^, *Sirpa*^wt/mut^ and *Sirpa*^wt/wt^ littermates had similar levels of disease-associated PrP deposits, as assessed by both immunohistochemistry and Western blotting, suggesting the existence of redundant or SIRPα-independent mechanisms for PrP^Sc^ clearance by microglia cells.

Collectively, our data show that all the pathological signs of prion diseases such as spongiosis, microglia activation and the accumulation of partially protease-resistant PrP were similar in all mice inoculated with prions, regardless of their *Sirpa* genotype, both in the cerebellum as well as in the forebrain. In light of these results, we can therefore conclude that SIRPα-mediated don’t eat me signaling is not a major determinant in prion disease pathogenesis in our analyzed mouse model.

Prions exist in many different strains and microglia activation is a strain-dependent phenomenon [54]. As such, we cannot exclude that SIRPα-dependent degradation processes may significantly impact on the prion pathogenesis in the context of other prion strains. Also, the mutant mice analyzed in the present study do express the extracellular part of SIRPα. It is formally possible that this may engage in molecular interactions (for example binding with CD47 on target cells, but possibly also with PrP^C^ or prions in the extracellular space) which could be of relevance for prion pathogenesis. If so, our study would have missed this effect. Again, the comparison of the recently developed conditional SIRPα knockout mouse with the mutant line will be instrumental to assessing the consequence of SIRPα ablation vs lack of SIRPα-mediated intracellular signaling.

As is the case for *Sirpa* in mice, the human orthologue *SIRPA* is also highly polymorphic [16]. The CD47-SIRPα pathway was recently shown to be a promising therapeutic target in the treatment of different types of tumors [25–27,55–57]. Further studies will be needed to explore whether *SIRPA* polymorphisms are clinically relevant, and whether CD47-SIRPα pathway may be a valid therapeutic target for a broader spectrum of diseases. In the case of prion diseases, the present results allow to exclude a major disease-modifying contribution by SIRPα and suggest the existence of additional, hitherto unidentified modulators of prion phagocytosis.

## Supporting information

S1 Fig Uncropped and unmodified Western blots of Fig 4A with size markers. (TIF)

S2 Fig Uncropped and unmodified Western blots of Fig 4B with size markers. (TIF)

S3 Fig Uncropped and unmodified Western blots of Fig 8B, upper panel, with size markers. (TIF)

S4 Fig Uncropped and unmodified Western blots of Fig 8B, lower panel, with size markers. (TIF)

S1 Table Clinical assessment and scoring of mice inoculated with rodent-adapted scrapie prions. (DOCX)

S2 Table Expression data (as reads per kilobase per million mapped reads, RPKM) of *Sirpa*, *Cd47*, *Aif1*, *Trem2* and *Mfge8* during prion disease as assessed by RNA sequencing. (XLSX)
