# Supplementary figures and images for: Prion pathogenesis is unaltered in the absence of SIRPα-mediated "don't-eat-me" signaling
Source: PLoS One. 2017 May 17;12(5):e0177876. doi: 10.1371/journal.pone.0177876 (PMC5435345; doi:10.1371/journal.pone.0177876)

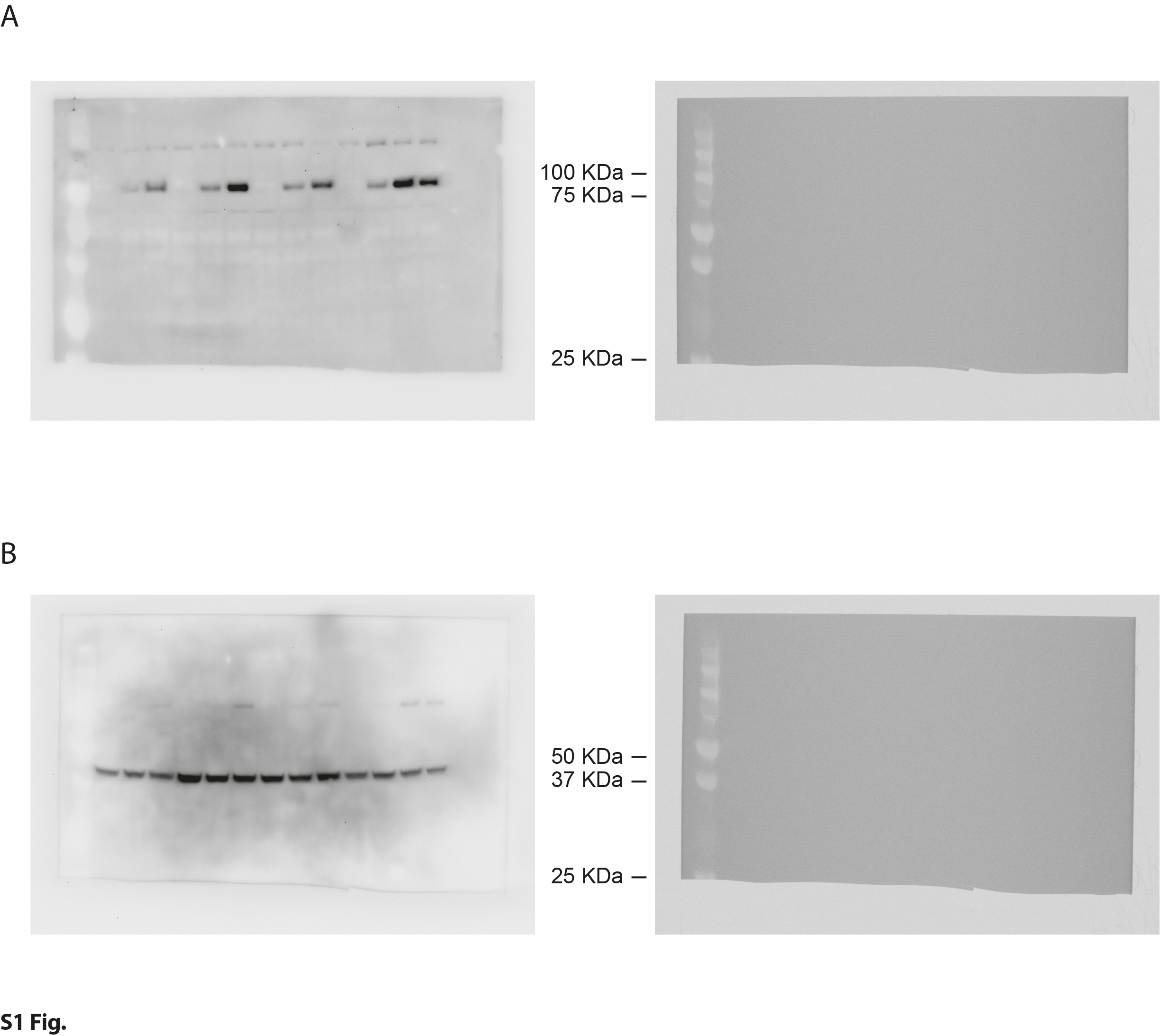

Supplement: S1 Fig — (TIF) [file pone.0177876.s001.tif]

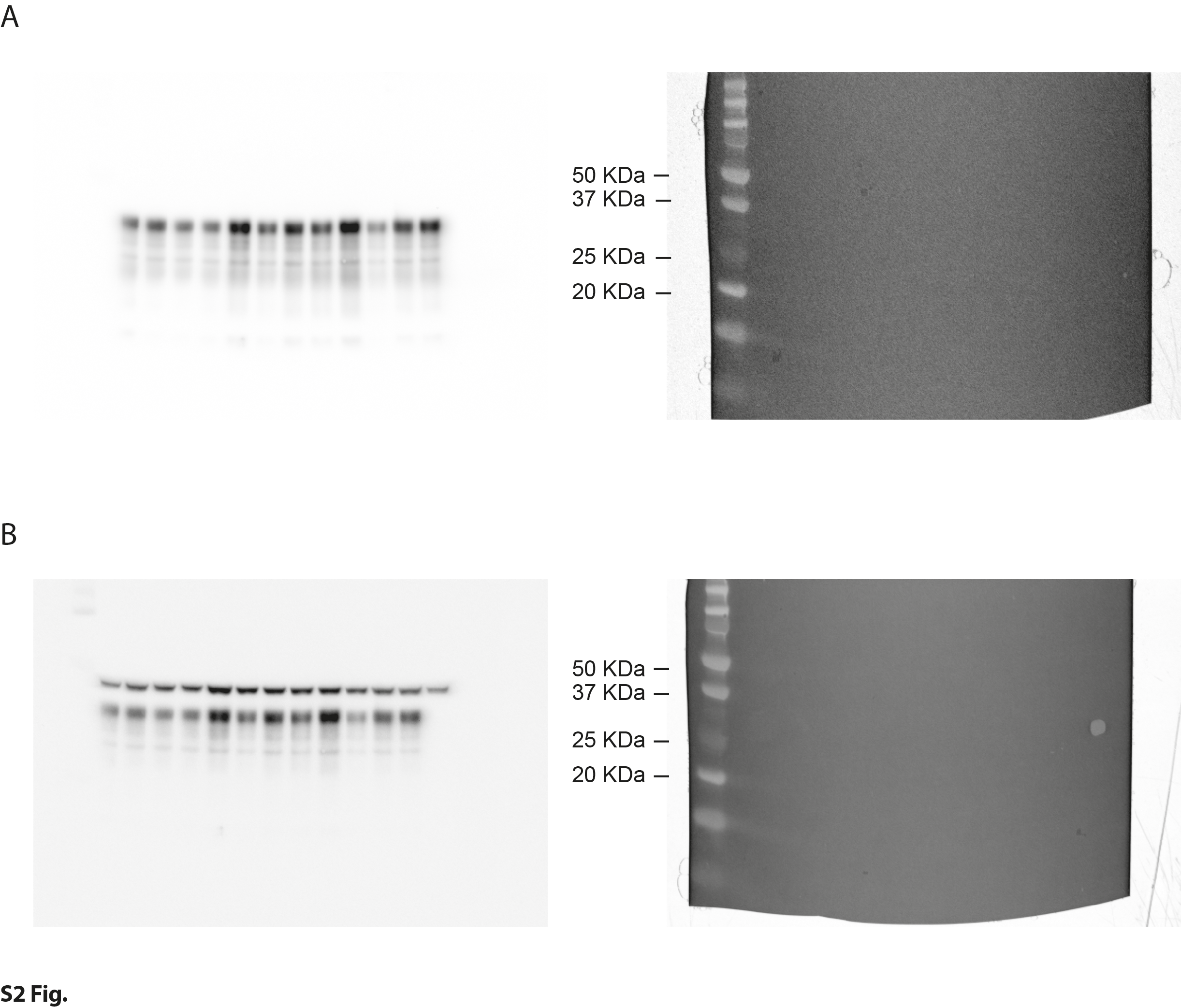

Supplement: S2 Fig — (TIF) [file pone.0177876.s002.tif]

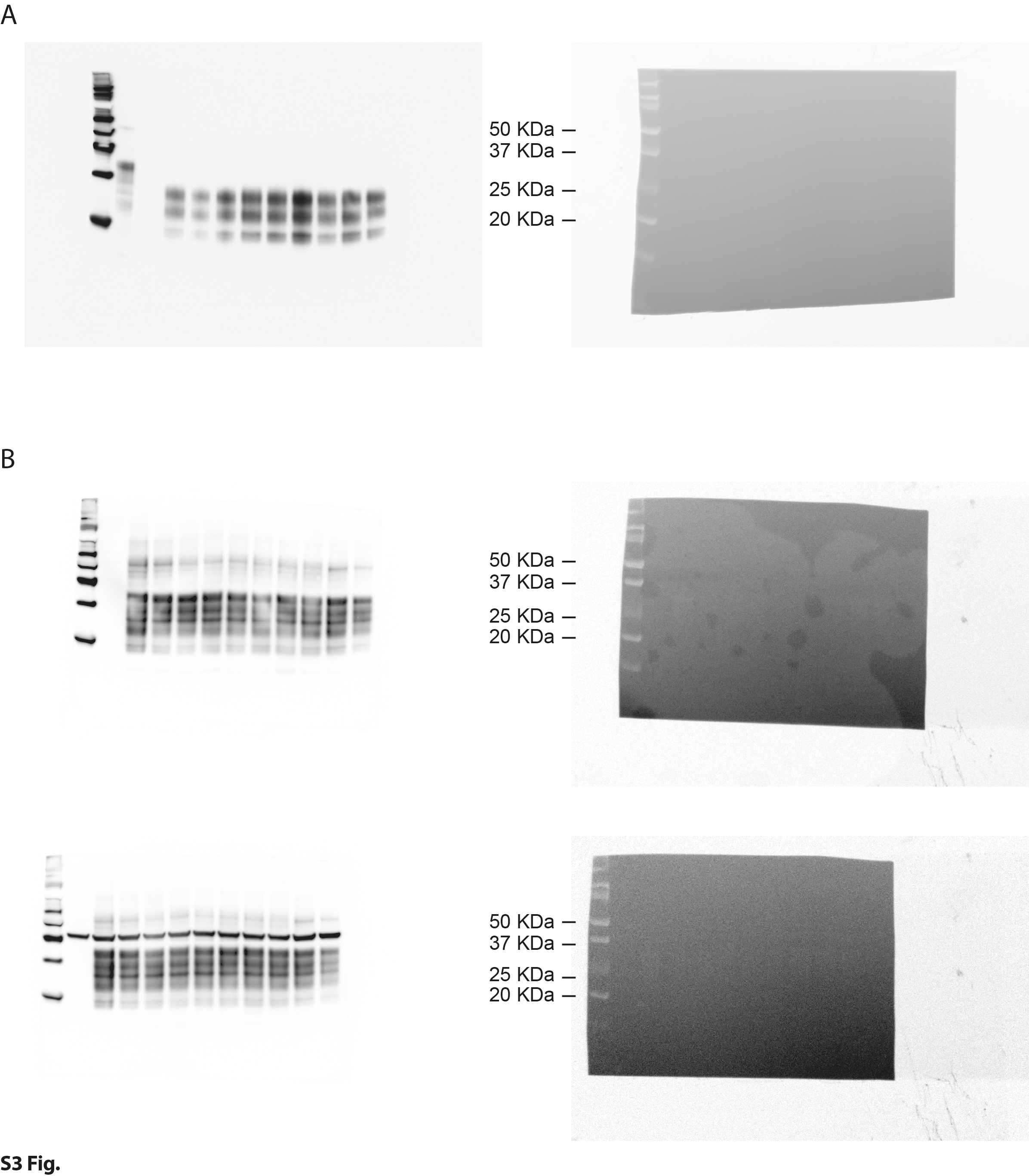

Supplement: S3 Fig — (TIF) [file pone.0177876.s003.tif]

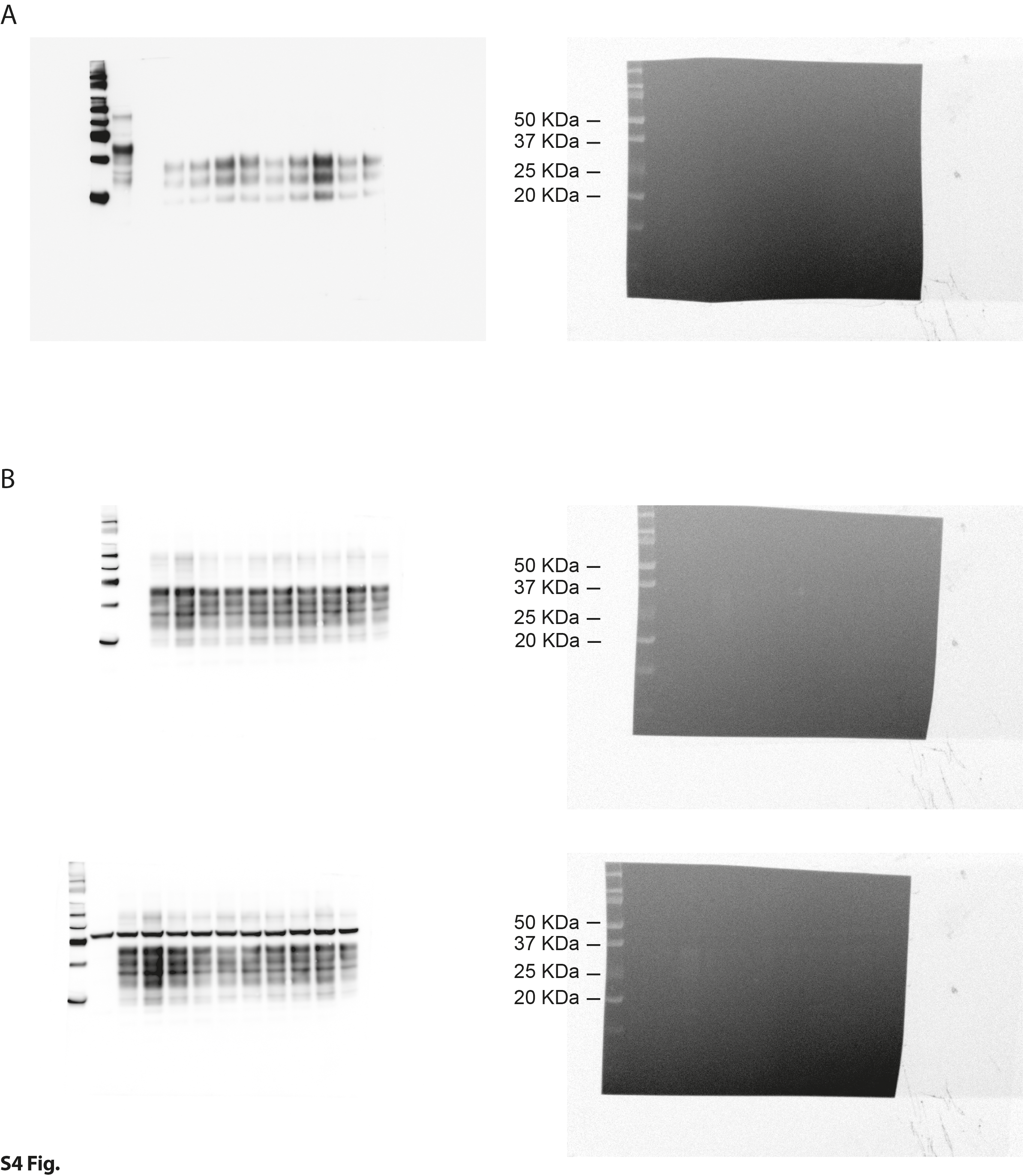

Supplement: S4 Fig — (TIF) [file pone.0177876.s004.tif]
