# Supplementary material for: Prion pathogenesis is unaltered in the absence of SIRPα-mediated "don't-eat-me" signaling
Source: PLoS One. 2017 May 17;12(5):e0177876. doi: 10.1371/journal.pone.0177876 (PMC5435345; doi:10.1371/journal.pone.0177876)
Supplement: S1 Table — (DOCX) [file pone.0177876.s005.docx]

**S1 Table. Clinical assessment and scoring of mice inoculated with rodent-adapted scrapie prions**

After inoculation, mice were observed three times per week for clinical signs including gait, grooming, activity, rough hair coat, limb paresis and ataxia. After the appearance of the first sign of scrapie (grade 1), mice were monitored every day and wet food was supplied in the cage. When mice reached score grade 2 that hamper the mice reaching water bottle, they were euthanized.

| **Score** | **Clinical signs** | **Assessment** | **Action** |
| --- | --- | --- | --- |
| 0 | No detectable signs of abnormal movement |  |  |
| 1 | Waddling gait, mild signs of reduced grooming, rough hair coat, limb weakness, front leg paresis | Slight rolling while shaking the cage | Provide wet food in the cage;  Observe every day |
| 2 | Ataxia, reduced grooming and activity, paralysis, rolling | Rolling while shaking the cage | Euthanize immediately if the clinical signs hamper the mice reaching the water bottle |
| 3 | Dead |  |  |

Reproduced from Nuvolone M et al. statin F is a biomarker of prion pathogenesis in mice. PLoS One 2017.
